# Evaluation of Whole-Genome Sequencing for Mycobacterial Species Identification and Drug Susceptibility Testing in a Clinical Setting: a Large-Scale Prospective Assessment of Performance against Line Probe Assays and Phenotyping

**DOI:** 10.1128/JCM.01480-17

**Published:** 2018-01-24

**Authors:** T. Phuong Quan, Zharain Bawa, Dona Foster, Tim Walker, Carlos del Ojo Elias, Priti Rathod, MMM Informatics Group, Zamin Iqbal, Phelim Bradley, Janet Mowbray, A. Sarah Walker, Derrick W. Crook, David H. Wyllie, Timothy E. A. Peto, E. Grace Smith

**Affiliations:** a The National Institute for Health Research (NIHR) Health Protection Research Unit in Healthcare Associated Infections and Antimicrobial Resistance, University of Oxford, Oxford, United Kingdom; b Nuffield Department of Medicine, University of Oxford, Oxford, United Kingdom; c Public Health England National Mycobacterial Reference Service, Birmingham, United Kingdom; d Wellcome Trust Centre for Human Genetics, University of Oxford, Oxford, United Kingdom; e National Infection Service, Public Health England, Colindale, London, United Kingdom; University of Iowa College of Medicine

**Keywords:** line probe assay, mycobacteria, phenotype, WGS, whole-genome sequencing

## Abstract

Use of whole-genome sequencing (WGS) for routine mycobacterial species identification and drug susceptibility testing (DST) is becoming a reality. We compared the performances of WGS and standard laboratory workflows prospectively, by parallel processing at a major mycobacterial reference service over the course of 1 year, for species identification, first-line Mycobacterium tuberculosis resistance prediction, and turnaround time. Among 2,039 isolates with line probe assay results for species identification, 74 (3.6%) failed sequencing or WGS species identification. Excluding these isolates, clinically important species were identified for 1,902 isolates, of which 1,825 (96.0%) were identified as the same species by WGS and the line probe assay. A total of 2,157 line probe test results for detection of resistance to the first-line drugs isoniazid and rifampin were available for 728 M. tuberculosis complex isolates. Excluding 216 (10.0%) cases where there were insufficient sequencing data for WGS to make a prediction, overall concordance was 99.3% (95% confidence interval [CI], 98.9 to 99.6%), sensitivity was 97.6% (91.7 to 99.7%), and specificity was 99.5% (99.0 to 99.7%). A total of 2,982 phenotypic DST results were available for 777 M. tuberculosis complex isolates. Of these, 356 (11.9%) had no WGS comparator due to insufficient sequencing data, and in 154 (5.2%) cases the WGS prediction was indeterminate due to discovery of novel, previously uncharacterized mutations. Excluding these data, overall concordance was 99.2% (98.7 to 99.5%), sensitivity was 94.2% (88.4 to 97.6%), and specificity was 99.4% (99.0 to 99.7%). Median processing times for the routine laboratory tests versus WGS were similar overall, i.e., 20 days (interquartile range [IQR], 15 to 31 days) and 21 days (15 to 29 days), respectively (*P* = 0.41). In conclusion, WGS predicts species and drug susceptibility with great accuracy, but work is needed to increase the proportion of predictions made.

## INTRODUCTION

In 2015, the World Health Organization (WHO) estimated that there were 10.4 million new tuberculosis (TB) cases, with 1.4 million deaths attributed directly to the disease (1). Three percent of new cases and 20% of previously treated cases are estimated to be resistant to one or more anti-TB drugs. Concerns over rises in the prevalence of multidrug-resistant TB (MDR-TB) strengthen requirements for rapid, effective diagnosis to identify drug resistance early, to target treatment effectively, and to reduce transmission. In this context, the WHO recommends molecular diagnosis by use of line probe assays (LPAs) (using PCR followed by hybridization) when multidrug resistance is suspected. A recent meta-analysis reported a high sensitivity and specificity of LPAs for detection of MDR-TB (2). In particular, the Hain GenoType MTBDR*plus* V1 and V2 assays have a sensitivity and specificity of 90% and 98%, respectively, for detection of rifampin resistance and 89% and 99%, respectively, for detection of isoniazid resistance (3).

There are 176 mycobacterial species (other than M. tuberculosis) published and available online (http://www.bacterio.net/mycobacterium.html), with Mycobacterium alsense being the most recent addition (added in 2016) (4). Given the increasing incidence of pulmonary nontuberculous mycobacterial (NTM) infections (5, 6), correct identification of NTM infections versus TB is becoming crucial for the management of clinical symptoms and treatment (7) (since NTM infections are tested and treated with a range of agents different from those used for TB), as well as being important for the public health response. Recent outbreaks of human disease arising from environmental contamination with M. chimaera (8) highlight the need to understand the role of environmental reservoirs of NTM more fully and to require accurate identification of NTM species isolated from clinical samples, particularly those cultured from nonpulmonary sites, such as blood and tissues.

Molecular tests can differentiate species more accurately than phenotypic tests, including biochemical tests, but multiple targets often exist, such as the 16S rRNA gene (9), 16S-23S rRNA internal transcribed spacer (10), and the beta subunit of RNA polymerase (encoded by *rpoB*) (11). The inability to discern differences between closely related mycobacterial species by use of one gene has led to use of multigene methods (12). The GenoType MTBC (Mycobacterium tuberculosis complex) and GenoType Mycobacterium CM/AS (common mycobacteria/additional species) assays (Hain Lifescience GmbH, Nehran, Germany) identify species via 23S rRNA gene PCR and reverse hybridization. The assays identify 6, 23, and 14 species of mycobacteria, respectively, either individually or by the combination of patterns detected. Concordance varies between the CM and AS tests (13, 14), with misidentification or lack of identification attributed to sequence variation in the probe region or ambiguous results. Evaluations of the GenoType Mycobacterium assays suggest an agreement with other methods of 89% (15).

Ongoing advances in whole-genome sequencing (WGS) of pathogens have the potential to significantly improve both the characterization of microbes, including mycobacteria, and investigations of suspected outbreaks. The use of WGS in mycobacterial diagnosis, including detection of multidrug-resistant M. tuberculosis isolates, is becoming a reality (16–19). Formal comparisons between WGS and standard molecular and other laboratory techniques are therefore essential if WGS is going to become the diagnostic test of choice.

We previously reported a pilot study demonstrating the potential of WGS for diagnosis of mycobacterial infection (16). In the present study, we formally assessed the agreement between WGS and standard laboratory practice in terms of NTM/MTBC species identification, first-line TB resistance prediction, and turnaround time by parallel processing of 2,171 clinical isolates from a major mycobacterial reference service over the course of 1 year.

## MATERIALS AND METHODS

The National Mycobacterial Reference Service in Birmingham, United Kingdom, provides reference testing to determine the species of all mycobacterial cultures submitted by National Health Service (NHS) laboratories across the Midlands and the North, with drug susceptibility testing (DST) and strain typing for MTBC isolates. During the study, it served 26 hospitals across the Midlands, covering around 12 million people.

WGS was run in parallel against routine laboratory diagnostic workflows from April 2015 to March 2016. Material from liquid cultured clinical samples signaling positive in mycobacterial growth indicator tubes (MGIT tubes; Becton Dickinson) was processed using Hain Lifesciences line probe assays to identify species (using the GenoType CM test followed by the GenoType Mycobacterium MTBC or GenoType Mycobacterium AS test, if required) and, for MTBC isolates, to assay isoniazid/rifampin drug susceptibility (using the GenoType MTBDR*plus* test). MTBC isolates were also tested phenotypically with first-line drugs (isoniazid, rifampin, ethambutol, and pyrazinamide) following the manufacturer's recommended procedures for the MGIT 960 system. An aliquot was also removed from the MGIT tube and prepared for WGS as previously described (20). Sequencing was attempted a maximum of twice, and data were centrally processed using a semiautomated bioinformatics pipeline. Within the pipeline, species identification was conducted using Mykrobe v0.3 (21). Isolates were also mapped to a library of mycobacterial reference genomes, and resistance predictions were made for any isolates which mapped to the MTBC reference strain H37Rv by use of our previously validated mutation catalogue (22).

WGS results were not returned to clinicians, so individual patient management was not affected by the study. As the WGS platform was still under development while the study was ongoing, turnaround time was partially estimated (see below), but final species and susceptibility predictions were based on final algorithms at the end of the study. Any isolates included in algorithm development were excluded from the final results.

Isolates with discordant routine laboratory and WGS results were regrown and reprocessed by both methods, and the laboratory results were reviewed by an experienced microbiologist.

### Statistical methods.

For species identification, WGS and LPA results were compared by binomial exact tests, taking the latter as the gold standard. Overall accuracy estimation was based on the following clinically important species (i.e., those identified at least annually from human clinical samples or closely related species): M. tuberculosis, M. africanum, M. bovis, M. bovis strain BCG, M. avium, M. chelonae, M. abscessus, M. fortuitum, M. gordonae, M. intracellulare, M. kansasii, M. malmoense, M. marinum, M. ulcerans, and M. xenopi. In cases where the line probe assays used were known to be unable to distinguish between certain species (i.e., *M. chelonae/M. immunogenum*, *M. intracellulare/M. chimaera*, *M. fortuitum/M. mageritense*, *M. malmoense/M. palustre*, and *M. peregrinum/M. septicum*), the results were considered concordant if WGS identified either species in the pair. Other rare species were reported separately. Mixtures were also reported separately, but the results were considered concordant if any species identified by LPA was also identified by WGS.

For TB drug resistance results, comparisons were made in two ways: (i) by comparing WGS identification of resistance-conferring mutations in the specific genes probed by the MTBDR*plus* test, i.e., *inhA* and *katG* for isoniazid resistance and *rpoB* for rifampin resistance, and (ii) by comparing the overall WGS resistance prediction against the phenotypic DST result. Comparisons used binomial exact tests, with the routine laboratory results considered the gold standard for both. MTBDR*plus*-identified resistance was also compared to the phenotypic DST result.

Turnaround time for the routine laboratory was measured from the date the automated liquid culture flagged positive to the date the complete set of susceptibility phenotypes for first-line drugs was reported. For WGS, the start of the process was considered to be the date the aliquot was taken from the MGIT tube. The WGS workflow consists of four main steps: extraction of DNA, sequencing of isolates (in batches), download of data from the local sequencing machine to the central bioinformatics pipeline (manually triggered), and processing of data in the pipeline to produce the clinical report. Since the WGS infrastructure was substantially upgraded partway through the study, turnaround time was measured up to the date the sequencing data arrived in the bioinformatics pipeline, and then the typical processing time of the new pipeline was added. Comparisons were made using the Wilcoxon signed-rank test.

### Accession number(s).

The sequences reported in this paper have been deposited in the NCBI Sequence Read Archive under BioProject number PRJNA401515.

## RESULTS

**FIG 1:**
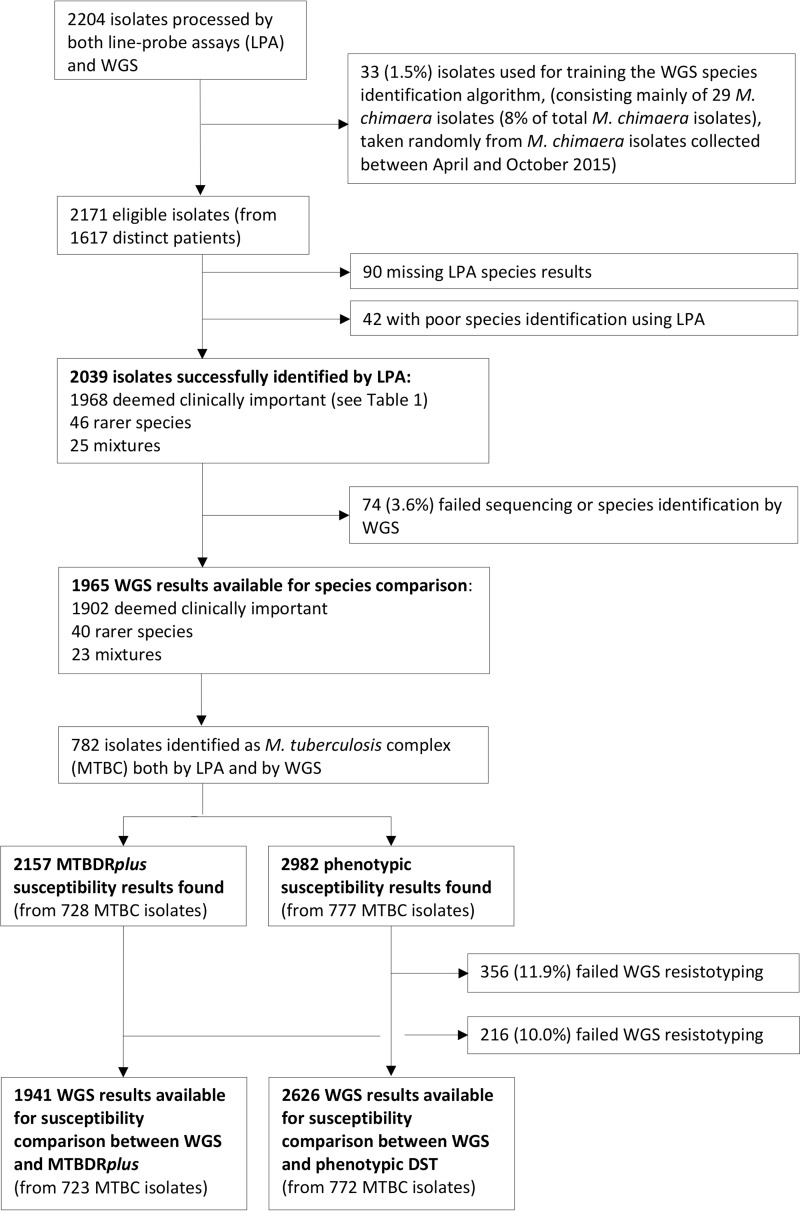
Isolates included in the study, identified by MGIT culture between 20 April 2015 and 31 March 2016.

Between 20 April 2015 and 31 March 2016, 2,204 liquid mycobacterial cultures of clinical samples which signaled positive for mycobacterial growth at the Public Health England National Mycobacterial Reference Service, Birmingham, United Kingdom, were split by volume and processed both by conventional tests and by WGS. Of these, 33 isolates were used for WGS species algorithm training, and the remaining 2,171 isolates (from 1,617 distinct patients) were eligible for inclusion in the study (Fig. 1). A total of 2,039 (93.9%) isolates were identified to the species or complex level by the routine laboratory (using the GenoType MTBC, GenoType Mycobacterium CM, and GenoType Mycobacterium AS line probe assays), and of these, 74 (3.6%) failed sequencing or WGS species identification (using Mykrobe v0.3 [21]), leaving 1,965 isolates available for direct species comparison.

**FIG 2:**
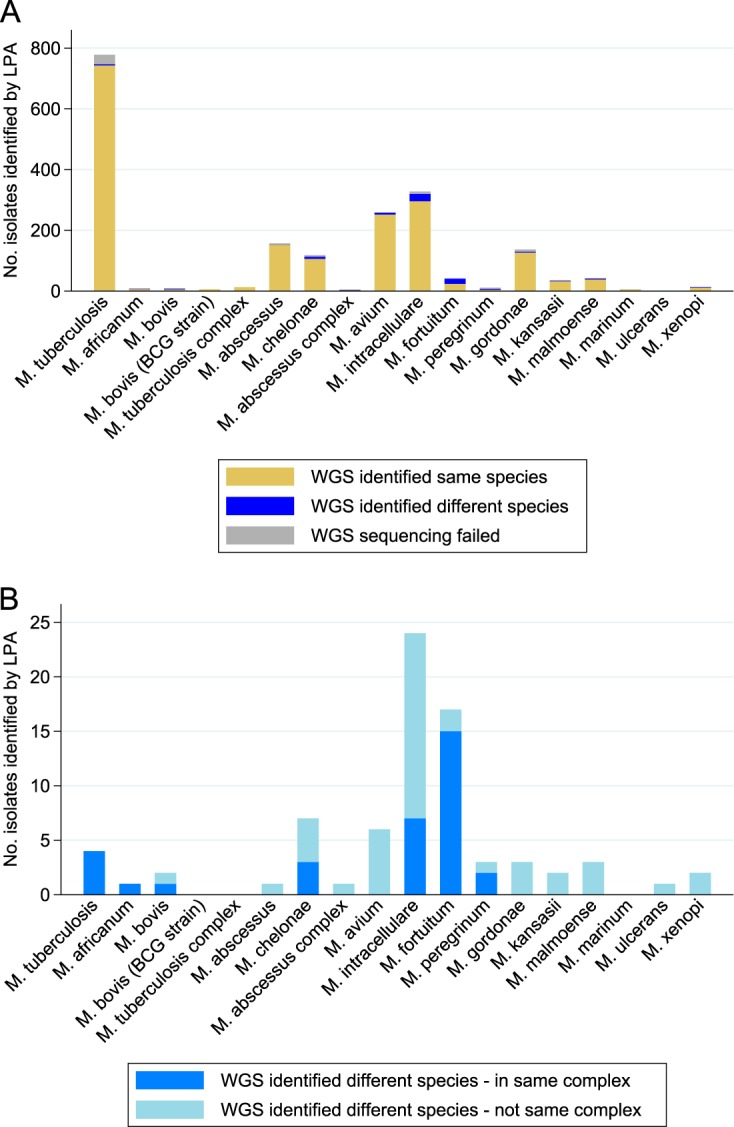
WGS species prediction versus line probe assay results. The bars represent the species identified by the line probe assay. (A) Concordance of WGS prediction at the species level. In cases where the line probe assays used were known to be unable to distinguish between certain species (i.e., *M. chelonae/M. immunogenum*, *M. intracellulare/M. chimaera*, *M. fortuitum/M. mageritense*, *M. malmoense/M. palustre*, and *M. peregrinum/M. septicum*), the results were considered concordant if WGS identified either species in the pair. (B) Isolates for which WGS identified a species different from that identified by the LPA.

A total of 1,968 of the 2,039 isolates were identified by LPA as clinically important mycobacterial species (Table 1), 46 were rarer species, and 25 were identified as mixtures. A total of 1,902 of the 1,968 clinically important isolates had a WGS comparator. Of the clinically important isolates, 1,825 (96.0% if excluding sequencing failures and 92.7% if including sequencing failures) were identified as the same species by WGS (Table 1; Fig. 2). Of the 77 cases where WGS identified a different species, 33 (42.9%) isolates were identified as a different species within the same complex (6 MTBC, 3 M. abscessus complex, 7 M. avium complex, and 17 M. fortuitum complex isolates). In 26 cases, WGS identified a species not probed for by the Mycobacterium CM/AS tests (Table 2). Fourteen of the discordant isolates were later found to have poor sequence quality, e.g., low numbers of reads, low coverage of the reference genome, or a GC content outside the range for mycobacteria. Detailed results for rarer species and mixtures can be seen in Table S1 in the supplemental material.

**TABLE 1:** WGS species predictions compared to those of line probe assays

Organism identified by GenoType MTBC, GenoType Mycobacterium CM, and GenoType Mycobacterium AS LPAs	No. of isolates identified by LPA	No. (%) of isolates that failed WGS sequencing or identification	No. of isolates for which WGS identified the same species^*a*^ (% excluding failures/% including failures)	No. of isolates for which WGS identified different species	
				In same complex	Not in same complex
M. tuberculosis complex organisms					
M. tuberculosis	778	31 (4.0)	743 (99.5/95.5)	4	0
M. africanum	8	0 (0.0)	7 (87.5/87.5)	1	0
M. bovis	8	0 (0.0)	6 (75.0/75.0)	1	1
M. bovis strain BCG	6	0 (0.0)	6 (100.0/100.0)	0	0
M. tuberculosis complex	13	0 (0.0)	13 (100.0/100.0)	0	0
Total	813	31 (3.8)	775 (99.1/95.3)	6	1
M. abscessus complex organisms					
M. abscessus	157	4 (2.5)	152 (99.3/96.8)	0	1
M. chelonae	118	5 (4.2)	106 (93.8/89.8)	3	4
M. abscessus complex	5	1 (20.0)	3 (75.0/60.0)	0	1
Total	280	10 (3.6)	261 (96.7/93.2)	3	6
M. avium complex organisms					
M. avium	258	0 (0.0)	252 (97.7/97.7)	0	6
M. intracellulare	328	8 (2.4)	296 (92.5/90.2)	7	17
Total	586	8 (1.4)	548 (94.8/93.5)	7	23
M. fortuitum complex organisms					
M. fortuitum	42	1 (2.4)	24 (58.5/57.1)	15	2
M. peregrinum	11	4 (36.4)	4 (57.1/36.4)	2	1
Total	53	5 (9.4)	28 (58.3/52.8)	17	3
Other nontuberculous mycobacteria					
M. gordonae	137	7 (5.1)	127 (97.7/92.7)		3
M. kansasii	36	2 (5.6)	32 (94.1/88.9)		2
M. malmoense	43	2 (4.7)	38 (92.7/88.4)		3
M. marinum	6	1 (16.7)	5 (100.0/83.3)		0
M. ulcerans	1	0 (0.0)	0 (0.0/0.0)		1
M. xenopi	13	0 (0.0)	11 (84.6/84.6)		2
Total	236	12 (5.1)	213 (95.1/90.3)		11
Total clinically important species	1,968	66 (3.4)	1,825 (96.0/92.7)	33	44
Rarer species^*b*^	46	6 (13.0)	11 (27.5/23.9)		
Mixtures^*c*^	25	2 (8.0)	21 (91.3/84.0)		

a In cases where the line probe assays used were known to be unable to distinguish between certain species (i.e., *M. chelonae/M. immunogenum*, *M. intracellulare/M. chimaera*, *M. fortuitum/M. mageritense*, *M. malmoense/M. palustre*, and *M. peregrinum/M. septicum*), the results were considered concordant if WGS identified either species in the pair.

b Rarer species include M. interjectum, M. scrofulaceum, M. genevense, M. goodii, M. lentiflavum, M. mucogenicum, M. simiae, and M. szulgai (see Table S1 in the supplemental material).

c Results for mixtures were considered concordant if WGS identified at least one of the species reported by line probe assay (Table S1).

**TABLE 2:** Clinically important isolates for which WGS identified a species different from that detected by LPA^*a*^

Initial LPA result	Initial WGS result	No. of isolates	No. of discrepancies resolved on repeat testing (no. that supported initial WGS results)	No. of results still discordant after repeat testing	No. of isolates that failed repeat testing (either LPA or WGS failure)
M. tuberculosis	M. africanum^*c*^	4	3 (2)	0	1
M. africanum	M. tuberculosis^*c*^	1	1	0	0
M. bovis	M. bovis strain BCG^*c*^	1	1	0	0
M. bovis	M. fortuitum	1	1	0	0
M. abscessus	M. avium	1	1 (1)	0	0
M. chelonae	M. tuberculosis	1	1	0	0
M. chelonae	M. abscessus^*c*^	3	1 (1)	0	2
M. chelonae	M. llatzerense^*b*^	2	1	1	0
M. chelonae	M. ratisbonense^*b*^	1	0	1	0
M. abscessus complex	M. llatzerense^*b*^	1	0	0	1
M. avium	M. tuberculosis	4	4	0	0
M. avium	M. chelonae	1	1	0	0
M. avium	M. shimoidei	1	1 (1)	0	0
M. intracellulare	M. tuberculosis	2	1	1	0
M. intracellulare	M. abscessus	1	0	0	1
M. intracellulare	M. avium^*c*^	1	0	0	1
M. intracellulare	M. tuberculosis and M. avium^*c*^	1	1	0	0
M. intracellulare	M. gordonae	1	1	0	0
M. intracellulare	M. arosiense^*b*^^,^^*c*^	1	0	1	0
M. intracellulare	M. colombiense^*b*^^,^^*c*^	2	0	2	0
M. intracellulare	M. marseillense^*b*^^,^^*c*^	2	0	2	0
M. intracellulare	M. paraffinicum	3	0	2	1
M. intracellulare	M. tomidae^*b*^	9	1	6	2
M. intracellulare	M. triplex^*b*^	1	0	1	0
M. fortuitum	M. chelonae	1	0	1	0
M. fortuitum	M. gordonae	1	0	0	1
M. fortuitum	M. peregrinum^*c*^	5	5 (5)	0	0
M. fortuitum	M. septicum^*c*^	5	0	4	1
M. fortuitum	M. farcinogenes^*b*^^,^^*c*^	1	0	1	0
M. fortuitum	M. porcinum^*b*^^,^^*c*^	4	0	4	0
M. peregrinum	M. kansasii	1	0	1	0
M. peregrinum	M. farcinogenes^*b*^^,^^*c*^	2	0	2	0
M. gordonae	M. tuberculosis	1	0	0	1
M. gordonae	M. avium	1	1 (1)	0	0
M. gordonae	M. intracellulare	1	0	0	1
M. kansasii	M. tuberculosis	1	1 (1)	0	0
M. kansasii	M. malmoense	1	1 (1)	0	0
M. malmoense	M. tuberculosis	1	1	0	0
M. malmoense	M. chimaera	1	0	0	1
M. malmoense	M. intracellulare	1	0	0	1
M. ulcerans	M. marinum	1	1 (1)	0	0
M. xenopi	M. abscessus	1	1 (1)	0	0
M. xenopi	M. tuberculosis and M. avium	1	1	0	0
Total		77	32 (15)	15	30

a Detailed results for the repeat tests can be seen in Table S2 in the supplemental material. Note that the M. tuberculosis complex includes M. tuberculosis, M. africanum, M. bovis, and M. bovis strain BCG, the M. abscessus complex includes M. abscessus and M. chelonae, the M. avium complex includes M. avium, M. intracellulare, M. chimaera, M. arosiense, M. colombiense, and M. marseillense, and the M. fortuitum complex includes M. fortuitum, M. mageritense, M. peregrinum, M. septicum, M. porcinum, and M. farcinogenes.

b Organism not in Mycobacterium CM/AS catalogue.

c Organism is in the same complex as the LPA-identified species.

Discordant isolates were retrieved from storage and retested by both methods. Thirty-two (42%) discordant cases were resolved (15 in which the LPA result changed, 16 in which the WGS result changed, and 1 in which both results changed), 30 (39%) cases remained discordant (2 in which the LPA result changed, 6 in which the WGS result changed, and 22 in which neither result changed), and 15 (19%) cases failed retesting (Table S2).

A total of 2,157 LPA results (obtained using GenoType MTBDR*plus*) for *inhA*, *katG*, and *rpoB* genes encoding resistance to the first-line drugs isoniazid and rifampin were available for 728 MTBC isolates. For DST, our WGS pipeline identifies point mutations by mapping isolates to an MTBC reference genome, and comparisons were made here by using *in silico* predictions based only on the specific codons targeted by the LPA. In 216 (10.0%) cases, WGS failed to make a prediction due to insufficient sequencing data. Excluding these cases, the overall concordance was 99.3% (95% confidence interval [CI], 98.9 to 99.6%), sensitivity was 97.6% (91.7 to 99.7%), and specificity was 99.5% (99.0 to 99.7%) (Table 3). Including the failures, the overall concordance was 89.4% (95% CI, 88.0 to 90.7%). By repeat testing of the 13 discordant isolates, four discordant cases were resolved (with the MTBDR*plus* result changing in all four cases), four cases remained discordant (one in which the MTBDR*plus* result changed and three in which neither result changed), and five isolates failed to regrow (Table S3). Of the four that remained discordant, two were because the MTBDR*plus* test identified the wild type while WGS consistently identified *rpoB* mutations encoding L430P and L452P changes. The remaining two instances of discordance can be considered “minor discordances,” as both assays identified mutations at the same codon (*rpoB*_H445), with the only difference being that the MTBDR*plus* result implied an amino acid mutation to aspartate (D) or tyrosine (Y), whereas the WGS results indicated the presence of leucine (L) at this site. All three amino acid substitutions are considered to cause resistance.

**TABLE 3:** WGS *in silico* LPA predictions compared to MTBDR*plus* predictions for MTBC specimens^*a*^

Gene (drug resistance)	No. of isolates															% failed	Sensitivity (95% CI)^*b*^ excluding failed isolates	Specificity (95% CI)^*b*^ excluding failed isolates	Overall % concordance (95% CI) excluding failed isolates	Overall % concordance (95% CI) including failed isolates^*c*^
	MTBDR*plus* MUT					MTBDR*plus* LWT					MTBDR*plus* WT									
	WGS prediction				Total	WGS prediction				Total	WGS prediction				Total					
	MUT	LWT	WT	F		MUT	LWT	WT	F		MUT	LWT	WT	F						
*inhA* (isoniazid)	17	0	1	1	19	0	0	1	0	1	0	0	652	48	700	6.8	94.4 (72.7–99.9)	100.0 (99.4–100.0)	99.7 (98.9–100.0)	92.9 (90.7–94.7)
*katG* (isoniazid)	47	0	0	6	53	0	0	0	0	0	3	0	621	44	668	6.9	100.0 (92.5–100.0)	99.5 (98.6–99.9)	99.6 (98.7–99.9)	92.6 (90.5–94.4)
*rpoB* (rifampin)	18	1	0	2	21	0	2	0	0	2	0	7	571	115	693	16.3	94.7 (74.0–99.9)	98.8 (97.5–99.5)	98.7 (97.4–99.4)	82.5 (79.6–85.3)
All	82	1	1	9	93	0	2	1	0	3	3	7	1,844	207	2,061	10.0	97.6 (91.7–99.7)	99.5 (99.0–99.7)	99.3 (98.9–99.6)	89.4 (88.0–90.7)

a MUT, mutation; LWT, loss of wild type; WT, wild type; F, failed WGS (insufficient sequencing data). If mutations were found at multiple sites in the same gene, MUT mutations were counted ahead of LWT mutations.

b Sensitivity and specificity relate to the ability of WGS to identify MTBDR*plus* MUT or WT results only, so any LWT WGS results were counted as discordant.

c Treating all failed results as discordant.

A total of 2,982 first-line phenotypic DST results (using the Becton Dickinson MGIT 960 system) for 777 MTBC isolates were available for the drugs isoniazid, rifampin, ethambutol, and pyrazinamide. This time, WGS predictions were made by comparing any mutations found to our previously validated mutation catalogue (22). In 356 (11.9%) cases, WGS failed to make a prediction due to insufficient sequencing data, and in 154 (5.2%) cases, the WGS prediction was indeterminate due to the presence of novel, previously uncharacterized mutations. Excluding these cases, the overall concordance was 99.2% (95% CI, 98.7 to 99.5%), sensitivity was 94.2% (88.4 to 97.6%), and specificity was 99.4% (99.0 to 99.7%) (Table 4). Including the failures and indeterminate predictions, the overall concordance was 82.2% (95% CI, 80.8 to 83.6%). In comparison, the overall concordance of the MTBDR*plus* test and phenotypic isoniazid/rifampin DST was 98.8% (98.1 to 99.3%), sensitivity was 84.0% (75.3 to 90.6%), and specificity was 100.0% (99.7 to 100.0%) (Table S4). Discordant isolates were not retested.

**TABLE 4:** WGS resistance predictions for MTBC specimens compared to phenotypic DST results^*a*^

Drug	No. of isolates										% uncharacterized/failed isolates		Sensitivity (95% CI) excluding U/F isolates	Specificity (95% CI) excluding U/F isolates	Overall % concordance (95% CI) excluding U/F isolates	Overall % concordance (95% CI) including U/F isolates^*c*^
	Phenotypically resistant					Phenotypically sensitive					U	F				
	WGS prediction				Total	WGS prediction				Total						
	R	S	U	F		R	S	U	F							
Isoniazid	67	5	0	9	81	0	572	35	64	671	4.7	9.7	93.1 (84.5–97.7)	100.0 (99.4–100.0)	99.2 (98.2–99.7)	85.0 (82.2–87.5)
Rifampin	28	0	0	3	31	2	586	20	118	726	2.6	16.0	100.0 (87.7–100.0)	99.7 (98.8–100.0)	99.7 (98.8–100.0)	81.1 (78.1–83.8)
Ethambutol	9^*b*^	0	0	0	9	9	574	92	68	743	12.2	9.1	100.0 (66.0–100.0)	98.5 (97.1–99.3)	98.5 (97.1–99.3)	77.5 (74.4–80.5)
Pyrazinamide	9	2	1	2	14	3	606	6	92	707	1.0	13.0	81.8 (48.2–97.7)	99.5 (98.6–99.9)	99.2 (98.1–99.7)	85.3 (82.5–87.8)
All first-line drugs	113	7	1	14	135	14	2,338	153	342	2,847	5.2	11.9	94.2 (88.4–97.6)	99.4 (99.0–99.7)	99.2 (98.7–99.5)	82.2 (80.8–83.6)

a R, resistant; S, sensitive; U, uncharacterized/indeterminate; F, failed WGS prediction (insufficient sequencing data to predict drug resistance).

b Includes two samples which were reported as phenotypically both resistant and susceptible.

c Treating all uncharacterized/failed results as discordant.

**FIG 3:**
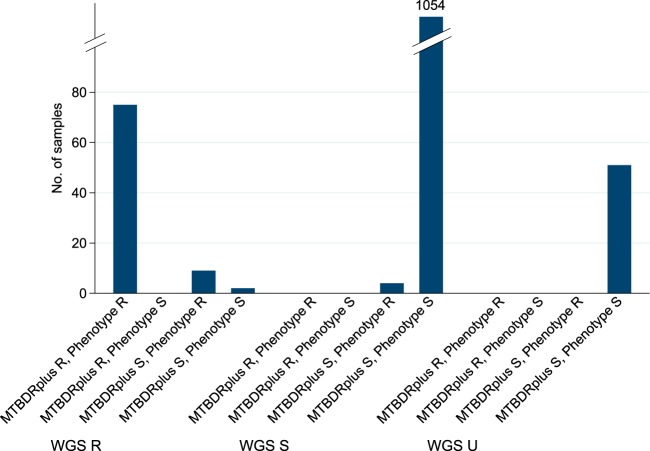
WGS susceptibility predictions versus those of MTBDR*plus* and phenotypic testing for the drugs isoniazid and rifampin. R, resistant; S, susceptible; U, uncharacterized mutations found. MTBDR*plus* results were treated as resistant if the result was mutation or loss of wild type. The MTBDR*plus* test does not claim to predict susceptibility (only the absence of known resistance mutations), but we labeled MTBDR*plus* “wild type” calls “susceptible” in order to aid comparisons.

A total of 1,183 WGS predictions had corresponding susceptibility results from both MTBDR*plus* testing and phenotypic DST for three-way comparison (Fig. 3). In all 51 cases where the WGS prediction was indeterminate (i.e., found only uncharacterized mutations), the isolate was phenotypically susceptible. In 9/11 (82%) cases where WGS predicted resistance and MTBDR*plus* testing did not, the phenotype was resistant (mutations found in phenotypically resistant isolates were *katG*_S315T [two times], *katG*_W328L [two times], *rpoB*_L452P [two times], *rpoB*_V170F [two times], and *rpoB*_H445L, and mutations found in phenotypically susceptible isolates were *rpoB*_L452P and *rpoB*_L430P; among these, MTBDR*plus* testing does not probe for *katG*_W328 or *rpoB*_V170 mutations). In the 4 cases where WGS predicted susceptibility and the phenotype was resistant, the MTBDR*plus* result did not indicate a resistance mutation either.

**FIG 4:**
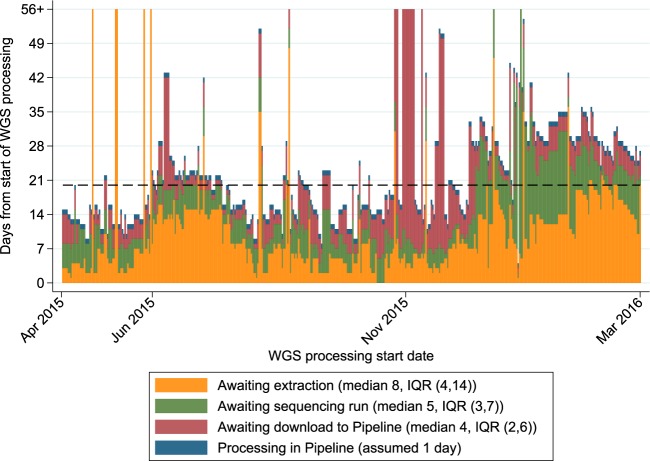
Differences in processing times for complete first-line susceptibility reporting for MTBC isolates. The horizontal line shows the median processing time for the routine laboratory, starting from the date that the MGIT tube was flagged as positive. The vertical lines show the different stages in the WGS process, starting from the removal of an aliquot from the positive MGIT tube. The WGS infrastructure was substantially upgraded partway through the study, with original report dates unavailable, so the typical processing time (1 day) of the new pipeline was used instead. Each line represents one isolate.

The turnaround time for all four first-line phenotypic DST results was available for 427 MTBC isolates. For these isolates, the median processing time for the routine laboratory was 20 days (interquartile range [IQR], 15 to 31 days), similar to the median processing time for WGS (21 days) (IQR, 15 to 23 days) (*P* = 0.41). However, performance varied noticeably across the 1-year study period, with WGS being faster in the first 6 months (median difference, 4 days faster [IQR, −3 to +12 days]; *P* < 0.001) and slower in the last 6 months (median difference, 7 days slower [IQR, −15 to +7.5 days]; *P* = 0.009). There was substantial variation in the time taken for three of the main steps in the WGS workflow (extraction of DNA, sequencing of isolates [in batches], and download of data from the local sequencing machine to the central bioinformatics pipeline [manually triggered]), with delays occurring in different stages depending on the period (Fig. 4). At its fastest, the processing time for the laboratory was around 8 days.

## DISCUSSION

In this large prospective study, we showed that WGS can be performed routinely in a high-throughput laboratory and that when predictions are made, they achieve very high agreement with current diagnostic tests for both mycobacterial species identification (96.0%) and MTBC first-line drug resistance detection (99.3% versus LPA and 99.2% versus phenotypic testing). About half of the differences in species identification were resolved on repeat testing, suggesting that they were possibly caused by laboratory errors or mixtures. There were continued differences after repeat testing for species identified by line probe assays as M. fortuitum, M. peregrinum, M. intracellulare, or M. chelonae, suggesting that these particular species should be the focus of further work.

Nearly half (13/30 isolates) of the isolates with persistent differences between WGS and line probe-based species identification methods were identified by routine tests as M. fortuitum or M. peregrinum, both of which are members of the M. fortuitum complex, which contains 12 different species. In all these cases, WGS identified another species within the same complex (but not present in the LPA catalogues) either before or after the repeat test. The Mycobacterium CM test splits this complex into two groups and may not differentiate them well due to similarity between sequences in the target sites (12). More accurate identification of the members of this complex to the species level may become clinically important in the future (24, 25).

Fifteen of the isolates with persistently discordant results at the species level were identified by the routine laboratory as M. intracellulare. Five of these were identified by WGS as other species within the M. avium complex (not including M. chimaera, which was treated as concordant with M. intracellulare since the Mycobacterium CM test does not distinguish between the two), and a recent study found that the Mycobacterium CM test often misidentifies rare M. avium complex species as M. intracellulare (26). A further seven isolates were classified as *M. tomidae* by WGS. The strain on which this classification was based was obtained from the National Collection of Type Cultures catalogue and is incorporated in the Mykrobe species classifier (21), part of the WGS bioinformatics pipeline. It appears that this particular strain has been catalogued variously as M. intracellulare (27), M. szulgae (28), and M. neoaurum (ATCC 23069; DSM 43993), suggesting that it is probably not reliable enough to be used as an exemplar in future. However, WGS consistently mapped these seven discordant isolates (in a workflow stream separate from the Mykrobe classification) to M. intracellulare, suggesting that the routine laboratory result was correct.

The two persistently discordant isolates that routine tests identified as M. chelonae were identified as M. llatzerense and M. ratisbonense by WGS. To the best of our knowledge, M. ratisbonense has previously been reported only from sewage (29). Given the continuing discovery and sequencing of rare mycobacteria, species identification catalogues should be updated regularly. WGS is reliant on the completeness and accuracy of sequence databases, which should not automatically be assumed to be error-free; therefore, in practice, surprising results such as these should be treated with caution and potentially verified using an alternative method.

In cases where predictions were made, the agreement between line probe assay, phenotyping, and WGS results for first-line drug resistance predictions was remarkably high. Similar results were recently reported for 462 prospectively collected MTBC isolates in the New York State Reference Laboratory (19); however, that U.S. study did not use WGS to identify mycobacterial species beyond differentiating MTBC from NTM isolates. One question is how isolates that failed WGS (denoted “F” in Tables 3 and 4) or had previously uncharacterized mutations (“U”) should be considered in calculating concordance. It can be argued that excluding them introduces a bias in favor of WGS, but on the other hand, including them creates a bias against WGS because there are two types of error to consider here: making an incorrect prediction versus not making a prediction at all. Including failures and uncharacterized mutations as automatically discordant makes the two errors indistinguishable, when in practice they are not the same. The former error would cause a clinician to make an error in management, whereas the latter indicates a lack of certainty which can then be managed clinically, similarly to a “loss of wild type” prediction from the MTBDR*plus* test, or by further testing. Note that, in this study, all but one isolate with previously uncharacterized mutations were phenotypically susceptible, and our definition of “failure” was very strict, requiring complete information at every base previously associated with resistance in relevant resistance-associated genes. The fact that WGS is still a new and improving technology means that the proportion of failures is likely to decrease in the coming years.

The turnaround time compared to that for phenotypic DST was not as short as expected, though still comparable, but this was not a true “real-time” head-to-head comparison, since the WGS analysis platform was still under development while the study isolates were being collected and sequenced, and the WGS processing was secondary in priority to the routine laboratory workflow. Now that WGS is in routine clinical use in the same laboratory, the current estimate for WGS turnaround time starting from MGIT positivity (measured over 4 weeks in May 2017) is around 7 days (E. G. Smith, personal communication), compared to 15 days in the New York State Reference Laboratory study (19), which is still 9 days earlier than that for their conventional methods. WGS is clearly slower than Hain LPAs (which typically return results the same day for a positive MGIT isolate), but this will continue to improve as the process matures and may be cut dramatically further if sequencing directly from samples becomes a reality (30).

This study had several limitations. It was conducted at only a single site, but this site is a large reference laboratory receiving isolates from multiple hospitals, and we previously showed that the extraction and sequencing process can be followed successfully at 17 sites internationally (16). Test results from the routine laboratory workflow were missing for a small proportion of isolates, and not all the isolates which showed discordant results were able to be regrown and retested. However, there is no reason to believe that this would lead to bias toward WGS. Although the isolates with discordant results had been stored at −20°C for several months and the repeat tests were carried out on fresh subcultures rather than on the original cultures, nevertheless, around half the discrepancies were resolved on repeat testing, suggesting that laboratory errors, intrinsic variability, or the presence of nonmycobacterial DNA in the original culture were responsible for much of the initial discordance. We did not carry out a cost analysis as part of this study but have shown previously that WGS-based diagnostics can actually be more cost-effective than routine workflows for a mycobacterial reference center (16).

As WGS is still a fairly new technology, it should be expected that some aspects of its implementation will need improvement, in particular increasing the proportion of predictions made as well as highlighting results that require further interpretation or repeat testing. The WGS predictions are deliberately conservative but can be refined further as more data accrue, particularly with respect to characterizing the effects of novel, potentially resistance-conferring mutations on DST predictions. As more NTM strains are sequenced, the phylogeny and relationships between species may change, along with their associations with clinical disease (23) and their antibiotic resistance profiles (31, 32). However, when predictions are made by WGS, they are very accurate, and as the rate of NTM infections rises, the increased information that WGS offers can only become more valuable.

## Supplementary Material

Supplemental material
