# Supplementary material for: Evaluation of Whole-Genome Sequencing for Mycobacterial Species Identification and Drug Susceptibility Testing in a Clinical Setting: a Large-Scale Prospective Assessment of Performance against Line Probe Assays and Phenotyping
Source: J Clin Microbiol. 2018 Jan 24;56(2):e01480-17. doi: 10.1128/JCM.01480-17 (PMC5786738; doi:10.1128/JCM.01480-17)
Supplement: Supplemental material [file supp_56_2_e01480-17__index.html]

Supplemental material 

# Evaluation of Whole-Genome Sequencing for Mycobacterial Species Identification and Drug Susceptibility Testing in a Clinical Setting: a Large-Scale Prospective Assessment of Performance against Line Probe Assays and Phenotyping

## Supplemental material

- Supplemental file 1 -

  Tables S1 (WGS species predictions compared to those obtained by line-probe assays for rarer species and mixtures), S2 (Repeat species tests for discordant samples), S3 (Repeat WGS *in silico* LPA predictions compared to those obtained by MTBDR*plus* for discordant samples), and S4 (LPA predictions compared to those obtained by phenotypic DST)

  PDF, 444K
