# Supplementary material for: Evaluation of Whole-Genome Sequencing for Mycobacterial Species Identification and Drug Susceptibility Testing in a Clinical Setting: a Large-Scale Prospective Assessment of Performance against Line Probe Assays and Phenotyping
Source: J Clin Microbiol. 2018 Jan 24;56(2):e01480-17. doi: 10.1128/JCM.01480-17 (PMC5786738; doi:10.1128/JCM.01480-17)
Supplement: Supplemental material [file JCM.01480-17_zjm999095810s1.pdf]

Table S1. WGS species predictions compared to line-probe assays for rarer species and mixtures

LPA result	WGS result	No. of isolates
M. interjectum	*M. intracellulare*	2
M. interjectum	*M. gordonae*	1
M. interjectum	*M. szulgai*	1
M. interjectum	Failed	1
M. scrofulaceum	*M. brisbanense**	1
M. scrofulaceum	*M. chimaera*	1
M. scrofulaceum	*M. chimaera;M. intracellulare*	1
M. scrofulaceum	*M. intracellulare*	1
M. genevense	*M. lentiflavum*	2
M. goodii	*M. smegmatis*	1
M. goodii	Failed	1
M. lentiflavum	*M. lentiflavum*	5
M. mucogenicum	*M. llatzerense**	11
M. mucogenicum	*M. ratisbonense**	5
M. mucogenicum	*M. tuberculosis;M. chimaera;M. intracellulare*	1
M. mucogenicum	Failed	3
M. simiae	*M. simiae*	2
M. szulgai	*M. szulgai*	4
M. szulgai	*M. angelicum**	1
M. szulgai	Failed	1
**Total rarer species**		**46**
M. tuberculosis; M. abscessus	*M. tuberculosis*	1
M. tuberculosis; M. avium	*M. tuberculosis; M. avium*	1
M. tuberculosis; M. avium	*M. tuberculosis*	1
M. tuberculosis; M. avium	*M. avium*	1
M. tuberculosis; M. chelonae	*M. tuberculosis*	1
M. tuberculosis; M. chelonae	*M. chelonae*	1
M. tuberculosis; M. intracellulare	*M. tomidae**	1
M. tuberculosis complex; M. avium	*M. avium*	1
M. tuberculosis complex; M. intracellulare	*M. tuberculosis*	1
M. abscessus; M. avium	*M. abscessus*	1
M. abscessus; M. fortuitum	*M. abscessus*	1
M. abscessus; M. intracellulare	*M. abscessus*	2
M. avium; M. intracellulare	*M. avium; M. intracellulare*	1
M. avium; M. intracellulare	*M. avium; M. chimaera*	1
M. avium; M. intracellulare	*M. avium*	1
M. avium; M. intracellulare	*M. chimaera*	1
M. avium; M. xenopi	*M. avium*	1
M. intracellulare; M. gordonae	*M. chimaera; M. gordonae*	1
M. intracellulare; M. gordonae	*M. chimaera*	1
M. intracellulare; M. gordonae	*M. gordonae*	1
M. intracellulare; M. xenopi	*M. chimaera*	1
M. intracellulare; M. xenopi	Failed	1
M. gordonae;M. kansasii	Failed	1
M. gordonae; M. xenopi	*M. tuberculosis*	1
**Total mixtures**		**25**

* Organism not in Mycobacterium CM/AS catalogue

Table S2. Repeat species tests for discordant samples

	No. of samples	Original species		Repeat species	
		LPA	WGS	LPA	WGS
Now concordant - LPA changed (n=15)					
	1	*M. abscessus*	*M. avium*	*M. avium*	*M. avium*
	1	*M. avium*	*M. shimoidei*	*M. shimoidei*	*M. shimoidei*
	1	*M. chelonae*	*M. abscessus*†	*M. abscessus*	*M. abscessus*
	5	*M. fortuitum*	*M. peregrinum*†	*M. peregrinum*	*M. peregrinum*
	1	*M. gordonae*	*M. avium*	*M. avium*	*M. avium*
	1	*M. kansasii*	*M. malmoense*	*M. malmoense*	*M. malmoense*
	1	*M. kansasii*	*M. tuberculosis*	*M. tuberculosis*	*M. tuberculosis*
	1	*M. tuberculosis*	*M. africanum*†	*M. africanum*	*M. africanum*
	1	*M. tuberculosis*	*M. africanum*†	*M. tuberculosis and M. africanum*	*M. africanum*
	1	*M. ulcerans*	*M. marinum*	*M. marinum*	*M. marinum*
	1	*M. xenopi*	*M. abscessus*	*M. abscessus*	*M. abscessus*
Now concordant - WGS changed (n=16)					
	1	*M. africanum*	*M. tuberculosis*†	*M. africanum*	*M. africanum*
	1	*M. avium*	*M. chelonae*	*M. avium*	*M. avium*
	4	*M. avium*	*M. tuberculosis*	*M. avium*	*M. avium*
	1	*M. bovis*	*M. bovis (BCG strain)*†	*M. bovis*	*M. bovis*
	1	*M. bovis*	*M. fortuitum*	*M. bovis*	*M. bovis*
	1	*M. chelonae*	*M. llatzerense**	*M. chelonae*	*M. chelonae*
	1	*M. chelonae*	*M. tuberculosis*	*M. chelonae*	*M. chelonae*
	1	*M. intracellulare*	*M. gordonae*	*M. intracellulare*	*M. intracellulare*
	1	*M. intracellulare*	*M. tuberculosis*	*M. intracellulare*	*M. chimaera*
	1	*M. intracellulare*	*M. tuberculosis and M. avium*	*M. intracellulare*	*M. chimaera*
	1	*M. malmoense*	*M. tuberculosis*	*M. malmoense*	*M. malmoense*
	1	*M. tuberculosis*	*M. africanum*†	*M. tuberculosis*	*M. tuberculosis*

	1	*M. xenopi*	*M. tuberculosis and M. avium*	*M. xenopi*	*M. xenopi*
Now concordant - Both changed (n=1)					
	1	*M. intracellulare*	*M. tomidae**	*M. intracellulare and M. avium*	*M. avium*
Still discordant - No change (n=22)					
	1	*M. chelonae*	*M. llatzerense**	*M. chelonae*	*M. llatzerense**
	1	*M. chelonae*	*M. ratisbonense**	*M. chelonae*	*M. ratisbonense**
	1	*M. fortuitum*	*M. farcinogenes*†*	*M. fortuitum*	*M. farcinogenes*†*
	3	*M. fortuitum*	*M. porcinum*†*	*M. fortuitum*	*M. porcinum*†*
	2	*M. fortuitum*	*M. septicum*†*	*M. fortuitum*	*M. septicum*†*
	1	*M. intracellulare*	*M. arosiense*†*	*M. intracellulare*	*M. arosiense*†*
	2	*M. intracellulare*	*M. colombiense*†*	*M. intracellulare*	*M. colombiense*†*
	2	*M. intracellulare*	*M. marseillense*†*	*M. intracellulare*	*M. marseillense*†*
	2	*M. intracellulare*	*M. paraffinicum*	*M. intracellulare*	*M. paraffinicum*
	6	*M. intracellulare*	*M. tomidae**	*M. intracellulare*	*M. tomidae**
	1	*M. intracellulare*	*M. triplex**	*M. intracellulare*	*M. triplex**
Still discordant - LPA changed (n=2)					
	2	*M. peregrinum*	*M. farcinogenes*†*	*M. fortuitum*	*M. farcinogenes*†*
Still discordant - WGS changed (n=6)					
	1	*M. fortuitum*	*M. chelonae*	*M. fortuitum*	*M. porcinum*†*
	1	*M. fortuitum*	*M. porcinum*†*	*M. fortuitum*	*M. tuberculosis*
	1	*M. fortuitum*	*M. septicum*†*	*M. fortuitum*	*M. avium*
	1	*M. fortuitum*	*M. septicum*†*	*M. fortuitum*	*M. tuberculosis*
	1	*M. intracellulare*	*M. tuberculosis*	*M. intracellulare*	*M. tomidae**
	1	*M. peregrinum*	*M. kansasii*	*M. peregrinum*	*M. farcinogenes*†*
Failed (n=15)					
	1	*M. abscessus/M. chelonae*	*M. llatzerense**	Failed	Failed
	2	*M. chelonae*	*M. abscessus†*	Failed	Failed
	1	*M. fortuitum*	*M. gordonae*	Failed	Failed
	1	*M. fortuitum*	*M. septicum*†*	*M. fortuitum*	Failed
	1	*M. gordonae*	*M. intracellulare*	Failed	Failed

	1	*M. gordonae*	*M. tuberculosis*	Failed	Failed
	1	*M. intracellulare*	*M. abscessus*	Failed	Failed
	1	*M. intracellulare*	*M. avium*†	*M. avium*	Failed
	1	*M. intracellulare*	*M. paraffinicum*	*M. intracellulare*	Failed
	1	*M. intracellulare*	*M. tomidae**	*M. intracellulare*	Failed
	1	*M. intracellulare*	*M. tomidae**	Failed	Failed
	1	*M. malmoense*	*M. chimaera*	Failed	*M. chimaera*
	1	*M. malmoense*	*M. intracellulare*	Failed	*M. intracellulare*
	1	*M. tuberculosis*	*M. africanum*†	Failed	*M. avium*

* Organism not in Mycobacterium CM/AS catalogue

† Organism is in same complex as LPA species

Notes:

The *M. tuberculosis* complex includes: *M. tuberculosis, M. africanum, M. bovis, M. bovis* (BCG strain)

The *M. abcessus* complex includes: *M. abcessus, M. chelonae*

The *M. avium* complex includes: *M. avium, M. intracellulare, M. chimaera, M. arosiense, M. colombiense, M. marseillense*

The *M. fortuitum* complex includes: *M. fortuitum, M. mageritense, M. peregrinum, M. septicum, M. porcinum, M. farcinogenes*

Table S3. Repeat WGS in-silico LPA predictions compared to MTBDR*plus,* for discordant samples. MUT = Mutation, LWT = Loss of wild type, WT = Wild type, F = Failed retesting.

	**No. of samples**	**Gene**	**Original result**		**Repeat result**	
			**MTBDR*plus***	**WGS**	**MTBDR*plus***	**WGS**
Now concordant - MTBDR*plus* changed (n=4)						
	1	*inhA*	LWT	WT	WT	WT
	2	*katG*	WT	MUT	MUT	MUT
	1	*rpoB*	WT	LWT	LWT	LWT
Still discordant - No change (n=3)						
	1	*rpoB*	MUT	LWT	MUT	LWT
	2	*rpoB*	WT	LWT	WT	LWT
Still discordant - MTBDR*plus* changed (n=1)						
	1	*rpoB*	WT	LWT	MUT	LWT
Failed (n=5)						
	1	*inhA*	MUT	WT	F	F
	1	*katG*	WT	MUT	F	F
	2	*rpoB*	WT	LWT	F	F
	1	*rpoB*	WT	LWT	F	LWT

Table S4. LPA predictions compared to phenotypic DST. MUT = Mutation, LWT = Loss of wild type, WT = Wild type, F = Failed to produce a clear result. Only includes samples where both a phenotypic and LPA result could be found.

	Phenotypically Resistant					Phenotypically Susceptible					Excluding failed (95% CI)		Overall concordance (95% CI)	
	LPA prediction				Total	LPA prediction				Total	Sensitivity*	Specificity*	Excluding failed	Including failed†
	MUT	LWT	WT	F		MUT	LWT	WT	F					
Isoniazid	63	0	10	1	74	0	0	618	0	618	86.3 (76.2-93.2)	100.0 (99.4-100.0)	98.6 (97.4-99.3)	98.4 (97.2-99.2)
Rifampicin	19	2	6	0	27	0	0	664	0	664	80.0 (57.7-91.4)	100.0 (99.4-100.0)	98.8 (97.7-99.5)	98.8 (97.7-99.5)
All	82	2	16	1	101	0	0	1282	0	1282	84.0 (75.3-90.6)	100.0 (99.7-100)	98.8 (98.1-99.3)	98.8 (98.0-99.3)

* For sensitivity and specificity calculations, all LWT LPA mutations were treated as Resistant, as this would usually be the clinical interpretation. MTBDR*plus* does not claim to predict susceptibility, (only absence of known resistance mutations), but we have treated WT calls as Susceptible in order to aid comparison.

† i.e. treating all failed results as discordant
